# Prevalence and incidence of treated schizophrenia: temporal and regional trends in Germany

**DOI:** 10.1038/s41537-025-00689-9

**Published:** 2025-11-05

**Authors:** Oliver Riedel, Christian J. Bachmann, Robert A. Bittner, Michael Dörks, Bianca Kollhorst, Mishal Qubad, Oliver H. F. Scholle

**Affiliations:** 1 Department of Clinical Epidemiology, Leibniz Institute for Prevention Research and Epidemiology – BIPS, Bremen, Germany; 2 Department of Child and Adolescent Psychiatry, Ulm University, Ulm, Germany; 3 Department of Psychiatry, Psychosomatic Medicine and Psychotherapy, Goethe University Frankfurt, University Medicine, Frankfurt, Germany; 4 Ernst Strüngmann Institute for Neuroscience (ESI) in Cooperation with Max Planck Society, Frankfurt, Germany; 5 Department of Health Services Research, Carl von Ossietzky University Oldenburg, Oldenburg, Germany; 6 Department of Biometry and Data Management, Leibniz Institute for Prevention Research and Epidemiology – BIPS, Bremen, Germany

**Keywords:** Schizophrenia, Psychosis

## Abstract

Schizophrenia ranks among the top ten causes of disability worldwide. The provision of healthcare services requires estimates on the epidemiology of schizophrenia, but recent data for Germany are lacking. Based on a large German health claims database (GePaRD), we identified persons aged 0–64 years with treated schizophrenia, i.e., persons having at least one inpatient/outpatient ICD-10 diagnosis (F20) with at least one prescription for a schizophrenia-recommended antipsychotic in the same calendar year. For each year from 2012 (eligible persons: 9,589,084) to 2021 (eligible persons: 12,450,531), we calculated the standardized incidence proportion (SIP) and the prevalence of schizophrenia. Analyses were stratified by sex, age, and population density in the region of residence. The SIP of treated schizophrenia remained stable from 2012 to 2017 (46.0–46.5/100,000) and subsequently declined to 41.3/100,000 in 2021, with higher SIP in men (45.3/100,000) than in women (37.1/100,000). In 2021, the SIP was comparable in urban, rural, and sparsely populated rural districts (36.3–38.5/100,000) and higher in large urban cities (48.3/100,000). SIP estimates among children and adolescents (aged 0–17 years) varied between 3.5/100,000 and 4.1/100,000 over the study period. The standardized prevalence of schizophrenia declined from 366.1/100,000 in 2012 to 334.0/100,000 in 2021. Similar to other Western countries, there has been a decline in the incidence and prevalence of schizophrenia in Germany over the last few years. The higher incidence in males and those living in large urban areas highlights the health care needs of these populations.

## Introduction

Schizophrenia is a severe mental disorder within the spectrum of psychotic disorders, characterized by positive symptoms such as hallucinations, delusions, and disorganized thinking, negative symptoms including flattened or inadequate affect and alogia as well as pervasive cognitive dysfunction. Patients are most often diagnosed in their twenties^1^. In rarer cases, the disorder can manifest before the age of 18 (early-onset schizophrenia, EOS) or even before the age of 13 commonly referred to as childhood-onset schizophrenia (COS)^2^. However, it is difficult to determine the exact onset of the disorder due to the often unspecific prodromal phase, resulting in a considerable duration of untreated psychosis (DUP), during which patients enter a more certain phase with more specific symptoms of the disorder without being adequately diagnosed and treated, for example with antipsychotics^3^.

Recently published findings from the Global Burden of Disease study reported an estimated prevalence of schizophrenia of 287/100,000 and an incidence of 16/100,000 in 2019, with mildly decreasing values of around 1 to 3% for both measures since 1990 and a stable male-female-ratio of 1.1^4^. Schizophrenia has also been reported to be associated with urbanicity as well as sociodemographic indices, with higher frequencies in populations of higher urbanicity and low socioeconomic status^5,6^.

Although the frequency of schizophrenia is low compared with other mental disorders, it nonetheless has a substantial impact on public health; it also creates a substantial socioeconomic burden for patients as well as their families and caregivers. The former is reflected in substantially reduced life expectancy with years of potential life lost globally estimated at 15.2 years^7^. Moreover, globally age-standardized disability-adjusted life years have been estimated at 184 per 100,000. For Europe, the direct annual costs of this mental disorder have been estimated at 29 billion euros^8,9^. Apart from the individual cases, a precise assessment of the frequency of the disorder is therefore of great importance from a socioeconomic perspective in order to estimate the need for treatment and optimize the provision of healthcare services. Health claims data are ideal for conducting such analyses, as they enable precise observations of patients across multiple healthcare sectors and over long periods of time. In addition, these data usually cover large numbers of individuals, which allows for robust estimates.

The aim of this study was to assess and characterize the incidence and prevalence of treated schizophrenia in Germany in children, adolescents, and adults (up to 64 years of age, to avoid misclassifications in higher age groups) across temporal and regional dimensions in a real-world setting and to describe those by urbanicity, socioeconomic status and sex.

## Methods

We conducted a population-based cross-sectional study, analyzing routinely collected German healthcare data.

### Data source

We used the so-called German Pharmacoepidemiological Research Database (GePaRD), which is based on claims data from four statutory health insurance (SHI) providers in Germany and currently includes information on ~25 million persons who were insured with one of the participating providers anytime since 2004^10^. Per data year, GePaRD covers ~20% of the general population and all geographical regions of Germany are represented. Demographic data include sex, age, and region of residence on the district level. Prescription data in GePaRD include all reimbursed drugs prescribed by general practitioners or specialists in the outpatient setting. Prescriptions are coded according to the German modification of the WHO Anatomical Therapeutic Chemical classification system (version from April 2023 for this study). In addition, GePaRD contains information on outpatient (i.e., from general practitioners and specialists) and inpatient services and diagnoses. Diagnoses are coded according to the German modification of the International Classification of Diseases and Related Health Problems, 10th Revision (ICD-10-GM).

### Study design

To be eligible for the year-wise study populations from 2012 to 2021, each person had to have (a) valid information on sex and an age between 0 and 64 years, (b) German residency, and (c) continuous insurance coverage in the respective year (gaps of up to 30 days were allowed). Persons who died or were born in the respective year were included but only required to be continuously insured from January 1 or until December 31, respectively, and were still considered in the full-year study population. For estimating the incidence, eligible persons additionally had to have both continuous insurance for the two years prior to the respective year and no history of treated schizophrenia (definition see below) during these two years. This 2-year washout period was applied uniformly across all study years, meaning that the same person could be classified as incident in multiple years if they again met these criteria.

### Identification of persons with schizophrenia

To enhance the validity of identifying individuals with schizophrenia, we used a more stringent approach than relying solely on coded diagnoses by focusing on persons with treated schizophrenia. A person was considered as treated for schizophrenia in a given year, if they had an in- or outpatient diagnosis of schizophrenia (ICD-10-GM code F20) in conjunction with at least one outpatient prescription of an antipsychotic considered potentially relevant for the treatment of schizophrenia (see Supplementary Table S1).

In addition to this main definition, we conducted two sensitivity analyses deviating from the primary approach by the following aspects. The first analysis considered only inpatient diagnoses of schizophrenia. In the second analysis, we included a broader range of diagnostic codes (both inpatient and outpatient) comprising schizophrenia spectrum disorders (ICD-10-GM codes F20, F21, F22, F23, F25, F28, and F29). As in the main analysis, subjects included in the sensitivity analyses were also required to have at least one prescription of the included antipsychotics in addition to the diagnostic codes considered.

### Regional characteristics

Via the person’s region of residence, we linked district-level characteristics (total number of districts: 401), including urbanicity and socioeconomic deprivation. Urbanicity was defined by the type of district according to settlement structure (as of 2017), categorized into four classes ranging from “large urban city” to “sparsely populated rural district”. Socioeconomic deprivation was measured using the German Index of Socioeconomic Deprivation (GISD; as of 2018). This index, developed by the Robert Koch Institute, quantifies regional socioeconomic deprivation in Germany by integrating indicators of education, employment, and income^11^.

### Data analysis

Separately for each year, we calculated the incidence proportion of schizophrenia, defined as the number of persons with newly identified schizophrenia who had not been identified as having schizophrenia in the two years before, per population (expressed as “per 100,000 persons”). We also calculated the prevalence of schizophrenia per population following the same methodology but omitting the two-year pre-observation period requirement.

Calculations were made overall and stratified by sex, age groups, and regional characteristics (only for the incidence). Persons younger than 18 years were considered for estimating EOS (13–17 years) and COS (younger than 13 years), respectively. Prevalence and incidence estimates were calculated along with 95% confidence intervals (CIs) and directly standardized according to age and sex with reference to the German population as of December 31, 2021. All statistical analyses were conducted using SAS version 9.4 (SAS Institute, Cary, NC, USA).

### Ethics

In Germany, the utilization of health insurance data for scientific research is regulated by the Code of Social Law. All involved health insurance providers as well as the Federal Office for Social Security and the Senator for Health, Women and Consumer Protection in Bremen as their responsible authorities approved the use of GePaRD data for this study. Informed consent for studies based on claims data is required by law unless obtaining consent appears unacceptable and would bias results, which was the case in this study. According to the Ethics Committee of the University of Bremen studies based on GePaRD are exempt from institutional review board review.

## Results

### Incidence overall and by sex and age

The number of eligible persons for estimating the incidence ranged between 9,589,084 (2012) and 12,450,531 (2021) (Supplementary Table S2). In 2021, the standardized incidence proportion was 41.3/100,000 overall and 45.3/100,000 in males and 37.1/100,000 in females (please refer to Supplementary Table S2 for the corresponding 95% CIs).

**Fig. 1:**
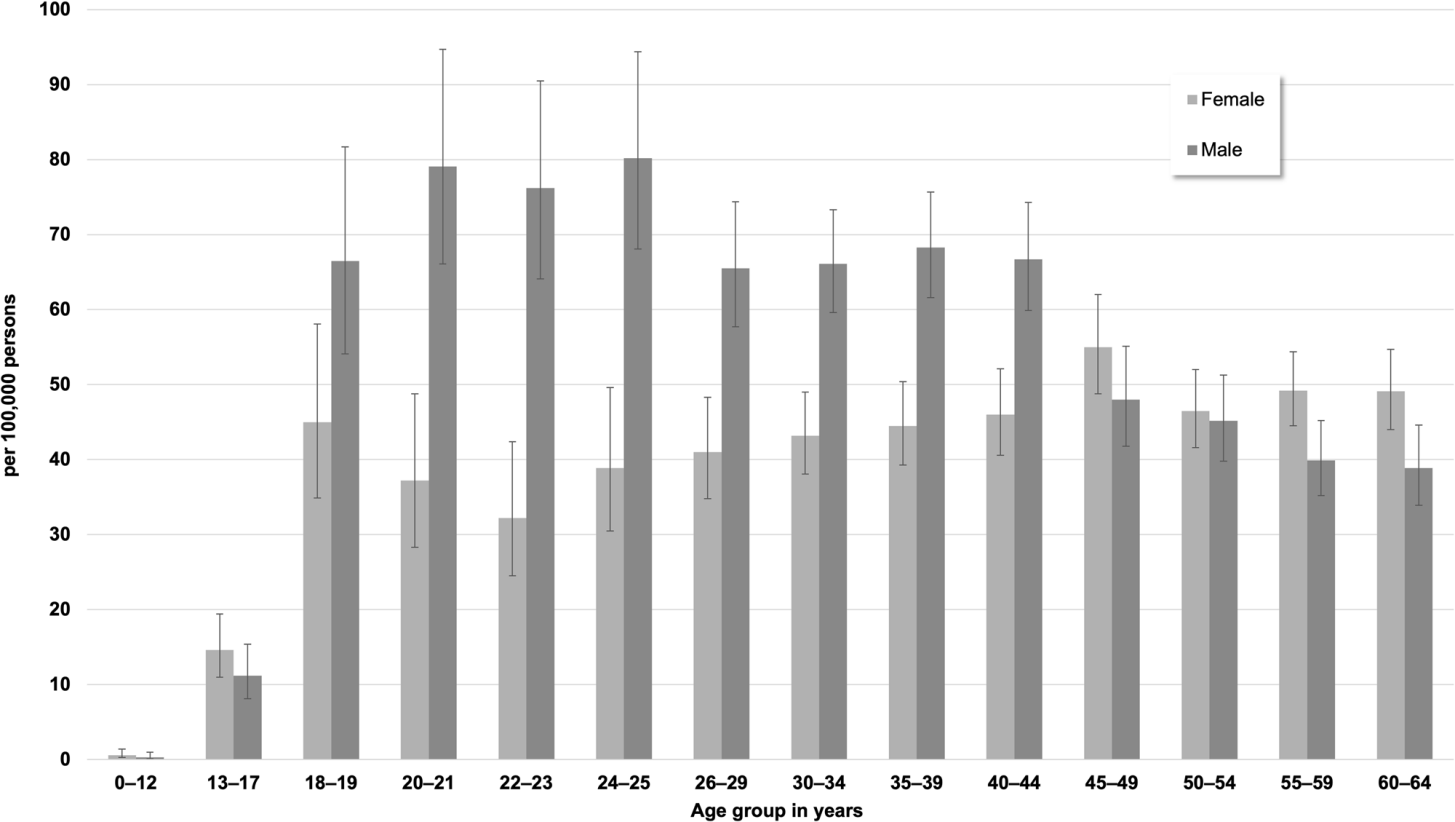
Standardized incidence proportions (with 95% CIs) of treated schizophrenia by sex and age in 2021 (main analysis = in-/outpatient ICD-10-diagnosis “F20” with antipsychotic treatments).

**Fig. 2:**
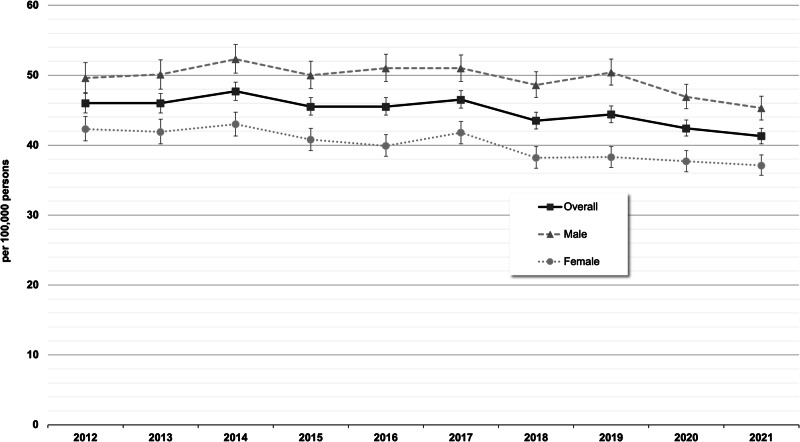
Standardized incidence proportions (with 95% CIs) of treated schizophrenia by sex and calendar year (main analysis = in-/outpatient ICD-10-diagnosis “F20” with antipsychotic treatments).

The standardized incidence in 2021 by sex and age is shown in Fig. 1 (see Supplementary Table S3 for absolute number of cases). In males, incidence proportions were highest in the age groups between 20 and 25 years (76.2–80.2/100,000); in females, the incidence proportions past the age of 18 years were similar across the age groups. Overall, the incidence remained stable in the years 2012–2017 (from 46.0–46.5/100,000), followed by a subsequent decline to 2021 (41.3/100,000; see Fig. 2 and Supplementary Table S2 for 95% CIs and see Supplementary Table S4 for absolute number of cases). Age- and sex-wise comparisons of the incidences between 2012 and 2021 indicated that the decline over the years was driven by a decline in the age groups 26–34 years among males and 30–64 years among females (see Supplementary Fig. S1).

The first sensitivity analysis, which considered only inpatient schizophrenia diagnoses, showed lower incidence proportions but similar age patterns (see Supplementary Fig. S2). Over time, a similar development to the main analysis was observed, with a stable overall incidence during 2012–2017 followed by a decline to 14.4/100,000 (95% CI: 13.7–15.0) in 2021 (see Supplementary Fig. S3 for a comparison of the overall standardized incidence proportions of schizophrenia according to the main analysis and the sensitivity analyses). The second sensitivity analysis using a broader range of schizophrenia spectrum disorders also showed a similar development over time but with higher incidence proportions (2021: 64.4/100,000, 95% CI: 63.0–65.8; see Supplementary Fig. S3).

**Fig. 3:**
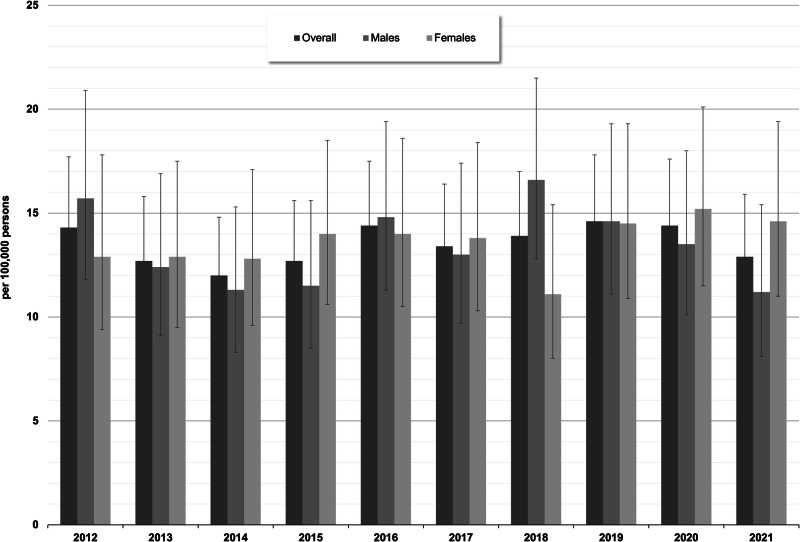
Standardized incidence proportions (with 95% CIs) of treated early-onset schizophrenia (age group 13–17 years) by sex and calendar year (main analysis = in-/outpatient ICD-10-diagnosis “F20” with antipsychotic treatments).

Regarding EOS, the overall incidence proportions varied between 12.0/100,000 (95% CI: 9.7–14.9) in 2014 and 14.6/100,000 (95% CI: 11.9–17.8) in 2019, with similar estimates for males and females and no clear temporal trend (see Fig. 3, see Supplementary Table S4 for absolute number of cases). For COS, no estimates are shown due to the low number of affected children (between 2 and 9 children per data year).

### Incidence by regional characteristics

**Fig. 4:**
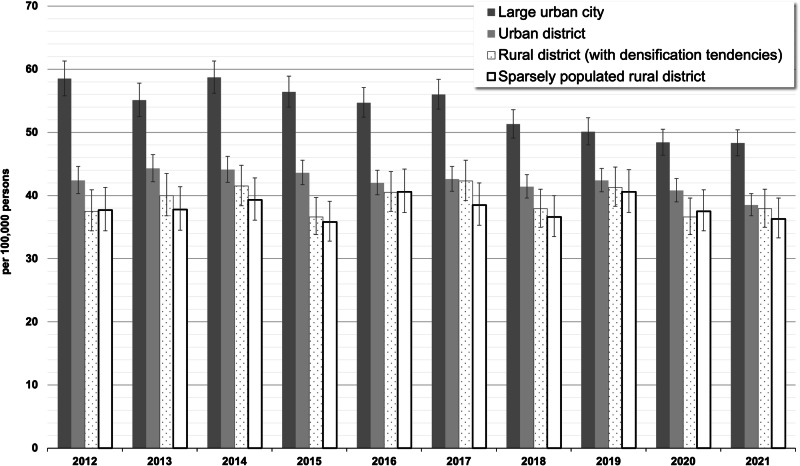
Standardized incidence proportions (with 95% CIs) of treated schizophrenia by urbanicity and calendar year (main analysis = in-/outpatient ICD-10-diagnosis “F20” with antipsychotic treatments).

Analyses stratified by urbanicity revealed a higher standardized incidence proportion in large urban cities (2021: 48.3/100,000) compared to urban districts, rural districts, and sparsely populated rural districts (36.3–38.5/100,000) (see Fig. 4, see Supplementary Table S4 for absolute number of cases). These differences in schizophrenia incidence were more pronounced in 2012 compared to 2021, primarily due to a 17% decrease in the incidence proportion in large urban cities over this period, along with unchanged or less pronounced decreases in the other classes of urbanicity.

The standardized incidence proportion of schizophrenia also differed across regional socioeconomic strata. In 2021, the 20% most deprived districts had a higher incidence of schizophrenia (48.6/100,000) compared to less deprived areas (60% moderately deprived districts: 40.8/100,000; 20% least deprived districts: 39.5/100,000; see Supplementary Fig. S4). The observed difference in 2021 developed over the course of the study period. Until the end of 2015, differences were minimal. From 2019 to 2021, however, schizophrenia incidence increased in 20% of the districts, namely those with the highest level of socioeconomic deprivation, while a decrease was seen in the two classes encompassing less deprived districts.

### Prevalence

**Fig. 5:**
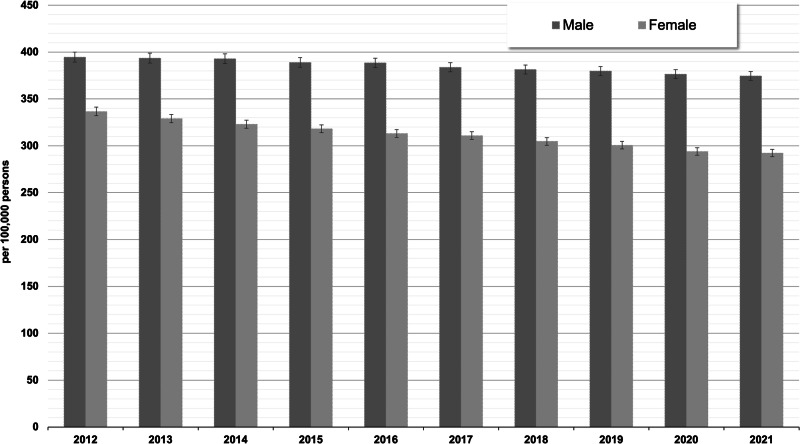
Standardized prevalence (with 95% CIs) of treated schizophrenia by sex and calendar year (main analysis = in-/outpatient ICD-10-diagnosis “F20” with antipsychotic treatments).

The number of eligible persons for estimating the prevalence ranged between 11,653,946 (2012) and 13,499,924 (2021). The overall prevalence of schizophrenia declined by 9% from 366.1/100,000 (95% CI: 362.6–369.6) in 2012 to 334.0/100,000 (95% CI: 331.0–337.1) in 2021, with higher prevalences in males than in females in each year (see Fig. 5, see Supplementary Table S4 for absolute number of cases). From 2012 to 2021, the relative decrease in the prevalence of schizophrenia was about twofold higher in women (–13%) compared to men (–5%).

## Discussion

We investigated the incidence and prevalence of schizophrenia from 2012 to 2021 in Germany and assessed differences by age, sex, and urban density of the region based upon health claims data. In 2021, we found an overall age- and sex-standardized incidence of schizophrenia of 41.3/100,000 when considering both outpatient and inpatient diagnoses. When including inpatient diagnoses only, the incidence decreased to 14.4/100,000.

The incidences of both definitions are consistent with epidemiological estimates that have been previously published. Based on systematic reviews, McGrath et al.^12^ reported a median incidence of 15.2/100,000, with 80% of estimates ranging between 7.7 and 43/100,000. Solmi et al.^4^ estimated an incidence for schizophrenia of 16.9/100,000 in 1990 and 16.3/100,000 in 2019, constituting a relative decline of 3.3% during that span. Of note, a decline was also seen in our data from 2012 to 2021, though with higher relative declines of 10% for the main definition and 22% for the inpatient definition. However, since neither study differentiated between inpatient and outpatient diagnoses, a direct comparison with our results is not feasible. It is possible that the decline in incidence in the last two years of our study period was at least partly influenced by the COVID-19 pandemic that occurred during this period, as the pandemic may have led to a general decline in contacts with routine care. However, the structure of the data we use is not designed to answer this research question, so these assumptions must remain speculative at this time.

Regarding the age- and sex-specific patterns of the incidences, we found highest incidence proportions in men between the age of 20 and 25 years, whereas in women, incidence proportions were lower and largely comparable across age groups over the age of 18 years. These sex-specific differences in the evolution of incidence proportions across age groups are well known. For example, our data resemble findings of a Finnish cohort study, which also reported a decline of incidences in men past the end of their twenties while in women the incidences are largely comparable across these age groups^13^. These findings dovetail with a recently published meta-analysis of 192 studies on the age of onset of mental disorders, which estimated a median age of onset of 25 years with an interquartile range of 21 to 35 years for schizophrenia^1^.

The large disparities between the incidences according to our main definition and the definition restricted to inpatient diagnoses might be surprising, assuming that most patients with incident schizophrenia initially require hospitalization. One potential explanation relates to the structure of German health care, which, among others, offers treatment conducted in specialized psychiatric facilities associated with hospitals, yet classified as outpatient facilities in the claims data. Nevertheless, we also assume that the disparities between the estimates can be attributed, at least in part, to the inclusion of less severe cases of schizophrenia in the main analysis due to the consideration of outpatient diagnoses. However, the incidence estimates from both definitions remain within the range of epidemiological estimates (as described above), and our objective was to estimate the frequency of treated schizophrenia in a real-world setting.

Among children and adolescents, the incidence of COS/EOS was between 3.3 and 4.1/100,000 in our study. This figure was lower than a previously reported incidence of 9/100,000 as estimated in a French cross-sectional assessment^14^. Their findings, however, have to be treated with caution due to methodological limitations regarding the low participation quota of patients and study centers. Regarding the overall incidence of EOS (i.e., among those aged 13–17 years), our findings are largely comparable to those presented by Okkels et al.^15^. Based on the Danish Psychiatric Central Research Register (DPCRR), which includes data from all admissions to psychiatric hospitals in Denmark since 1969, the authors estimated an incidence rate of 16/100,000 among those aged 12–18 years for the period of 1994 to 2010. However, there is still a considerable variance with regard to the estimated incidence of schizophrenia in younger age groups. Driver et al.^2^ reported from other studies, based on an NIMH cohort of patients below the age of 13 years, and stated that it is generally accepted that the incidence of COS is below 40/100,000. Recently published findings from a Finnish birth cohort study, which investigated cumulative incidences of non-affective psychotic disorders, found 0.2% of males and 0.7% of females to be diagnosed correspondingly below the age of 18 years, with 27% of those being diagnosed with schizophrenia^16^. Regarding these heterogeneous results, our own findings are difficult to interpret. It must also be borne in mind that, particularly in younger age groups, there is often a considerable symptomatic overlap with other mental disorders (e.g., autism spectrum disorders, developmental delay), which makes an exact diagnosis even more difficult^17–19^. In addition to that, Vernal et al.^20^ reported based on a register study that outpatient diagnoses of EOS can be of lower validity, which might also have contributed to our results. Furthermore, the DUP in patients with COS/EOS was considerably longer than in adult-onset schizophrenia (average DUP = 18.7 months in COS/EOS vs. 10.7 months in adult-onset schizophrenia^3^. This discrepancy might have contributed to an underestimation of COS/EOS cases. It is also conceivable that the exact diagnosis of schizophrenia may be given more reluctantly in younger people due to its implications and potential stigmatization, with diagnoses for broader nonaffective psychotic disorders being used instead. However, due to the design of our study, we cannot make any statements on this and suggest that case-finding algorithms be expanded accordingly in future, similar studies.

We found that the incidence of schizophrenia varied by urbanicity and regional socioeconomic strata. The higher incidence of schizophrenia in large urban cities aligns with prior research, which showed that individuals in the most urbanized environments exhibited more than double the risk of schizophrenia compared to those in the most rural areas^6^. Potential explanations for this association include environmental and social stressors, such as social fragmentation, material deprivation, and increased exposure to air pollution, all of which could contribute to heightened stress^21,22^. Another possible explanation is that stigmas associated with mental disorders may discourage individuals from seeking care in rural areas, or that rural physicians may be hesitant to formally code schizophrenia due to concerns about labeling individuals in a socially restrictive environment. The observed higher incidence of schizophrenia in districts with lower socioeconomic status supports previous findings that socioeconomic disadvantage is associated with an increased risk of schizophrenia^5,23^.

We found an overall age- and sex-standardized prevalence of 366/100,000 in 2012 and 334/100,000 in 2021, respectively, both of which are in line with previous findings. An early meta-analysis based on more than 150 studies reported a slightly higher prevalence rate of 460/100,000^12^. Simeone et al.^24^ found a median 12-month-prevalence of 330/100,000 (interquartile range: 260/100,000–510/100,000), based on a systematic review, while the earlier-mentioned GBD study estimated a prevalence of 287/100,000 in 2019^4^. As was the case for the incidence estimates, we also found a decline in the prevalence estimates of around 9% from 2010 to 2021, which again is higher than the decline as reported from the GDB study data (0.9%). Similarly, a recently published study on the prevalence of diagnostic codes for mental disorders reported a relative decline of 12% for schizophrenia (ICD-10 F20-F29) from 2012 to 2022^25^. However, these findings, though also based on German health claims data, have to be interpreted with caution, since the underlying study already counted the single occurrence of a code, while the algorithm established in our analyses was stricter to increase validity. The observed decline in administrative schizophrenia prevalence may also reflect features of the German health system; however, national schizophrenia guidelines, the ICD-10-GM coding system, and reimbursement policies did not change during the study period. Between 2012 and 2021 the number of practicing psychiatrists increased, and Germany’s population grew by roughly 1.5 million—mainly due to immigration—an increase that would ordinarily be expected to raise psychosis risk and thus prevalence. Conversely, population growth could have strained mental health services and left some cases undetected. Thus, overall the authors could not identify any specific changes in the German mental health system that clearly explain the falling schizophrenia prevalence.

### Strengths and limitations

The main strength of the present study is the underlying health claims database, which comprised data from a large proportion of the German population over a comparably long period of time. Both aspects enabled us to study temporal and regional trends of schizophrenia based on a large sample. Moreover, our findings potentially contribute to research on the epidemiology of EOS, which still can be considered understudied. Our chosen case definition of schizophrenia, which was based on in- and outpatient diagnoses in conjunction with specific prescriptions can also be regarded as highly valid. In fact, the consistency of both the estimated frequency of schizophrenia and the observed differences by urbanicity and district-level socioeconomic status with findings from prior epidemiological studies suggests that GePaRD is feasible for schizophrenia research.

However, in addition to the previously mentioned limitations, further flaws should be kept in mind when interpreting our findings. Firstly, our case definition was based on ICD-10 diagnoses in conjunction with antipsychotic treatment, thus not including patients with schizophrenia but no prescription of antipsychotics during the observation time. Although such cases are likely to occur only very rarely due to the severity of the disorder, they would not have been detected by our algorithm, potentially leading to underestimation of both incidence and prevalence. A further limitation is the lack of information regarding individual socioeconomic status as well as migration status, which have been previously identified as a risk factor^5,23,26,27^. Moreover, although GePaRD covers a substantial proportion of persons with SHI memberships each year, it should not necessarily be considered representative for the general population. While in Germany, citizens and permanent residents are obliged to have health insurance, ~10% of persons are members of private health insurance providers (e.g., due to an annual gross salary above a certain income threshold or due to belonging to specific occupational groups)^28^. These memberships are not covered by GePaRD. Finally, since our results are restricted to the age range under study, persons of 65 years or older were not considered. Although schizophrenia can also occur in higher ages, we wanted to avoid biases due to misclassifications (e.g., organic dementia syndromes with psychotic symptoms) and have therefore excluded older persons.

In conclusion, even when considering the slight declines in the frequencies of schizophrenia over the last years, our real-world data still highlight the public-health burden associated with this mental disorder. This is especially true for male patients and those living in large urban areas, which underscores the need for optimized care structures for these populations.

## Supplementary information

Supplementary Material_CLEAN

## Data Availability

As we are not the owners of the data, we are not legally entitled to grant access to the data of the German Pharmacoepidemiological Research Database. In accordance with German data protection regulations, access to the data is granted only to employees of the Leibniz Institute for Prevention Research and Epidemiology – BIPS on the BIPS premises and in the context of approved research projects. Third parties may only access the data in cooperation with BIPS and after signing an agreement for guest researchers at BIPS.
