# Supplementary material for: Prevalence and incidence of treated schizophrenia: temporal and regional trends in Germany
Source: Schizophrenia (Heidelb). 2025 Nov 5;11(1):131. doi: 10.1038/s41537-025-00689-9 (PMC12589479; doi:10.1038/s41537-025-00689-9)
Supplement: Supplementary file 1 — Supplementary Material_CLEAN [file 41537_2025_689_MOESM1_ESM.pdf]

SUPPLEMENTARY MATERIAL

Table S1: Antipsychotics used in this study for the identification of persons with treated schizophrenia

**Drug name**	**ATC code**	**Antipsychotic class**
Chlorpromazine	N05AA01	Typical
Triflupromazine	N05AA05	Typical
Cyamemazine	N05AA06	Typical
Fluphenazine	N05AB02	Typical
Perphenazine	N05AB03	Typical
Thiopropazate	N05AB05	Typical
Perazine	N05AB10	Typical
Thioridazine	N05AC02	Typical
Haloperidol	N05AD01	Typical
Trifluperidol	N05AD02	Typical
Bromperidol	N05AD06	Typical
Benperidol	N05AD07	Typical
Sertindole	N05AE03	Atypical
Ziprasidone	N05AE04	Atypical
Lurasidone	N05AE05	Atypical
Flupentixol	N05AF01	Typical
Zuclopenthixol	N05AF05	Typical
Fluspirilene	N05AG01	Typical
Pimozide	N05AG02	Typical
Clozapine	N05AH02	Atypical
Olanzapine	N05AH03	Atypical
Quetiapine	N05AH04	Atypical
Asenapine	N05AH05	Atypical
Olanzapine and samidorphan	N05AH53	Atypical
Sulpiride	N05AL01	Atypical
Amisulpride	N05AL05	Atypical
Risperidone	N05AX08	Atypical
Zotepine	N05AX11	Atypical
Aripiprazole	N05AX12	Atypical
Paliperidone	N05AX13	Atypical
Iloperidone	N05AX14	Atypical
Cariprazine	N05AX15	Atypical
Brexpiprazole	N05AX16	Atypical

Antipsychotic drugs included all types of dosage forms, including long-acting injectables.

Table S2: Standardized incidence proportion (with 95% CIs) of schizophrenia by demographic characteristics and calendar year (per 100,000 persons)

	**2012**	**2013**	**2014**	**2015**	**2016**
**Total number of (database) population, 0–64 years, n**	9,589,084	9,879,340	10,958,044	11,361,701	11,403,109
**Overall**					
Both sexes	46.0 (44.6; 47.4)	46.0 (44.7; 47.4)	47.7 (46.4; 49.1)	45.5 (44.3; 46.8)	45.5 (44.3; 46.8)
Females	42.3 (40.6; 44.1)	41.9 (40.1; 43.7)	43.0 (41.4; 44.8)	40.8 (39.3; 42.5)	39.9 (38.3; 41.5)
Males	49.6 (47.5; 51.7)	50.1 (48.0; 52.2)	52.3 (50.3; 54.4)	50.0 (48.1; 52.0)	51.0 (49.1; 53.0)
**Age group 0–17 years**					
Both sexes	4.1 (3.4; 5.1)	3.5 (2.9; 4.4)	3.7 (3.0; 4.5)	3.6 (2.9; 4.4)	4.1 (3.4; 5.0)
Females	3.7 (2.7; 5.0)	3.6 (2.7; 4.9)	3.6 (2.7; 4.9)	3.9 (2.9; 5.1)	3.8 (2.9; 5.1)
Males	4.6 (3.5; 6.0)	3.5 (2.6; 4.7)	3.7 (2.8; 4.9)	3.3 (2.5; 4.4)	4.4 (3.4; 5.7)
**Age group 18–64 years**					
Both sexes	57.4 (55.7; 59.2)	57.6 (55.9; 59.4)	59.7 (58.1; 61.4)	56.9 (55.4; 58.5)	56.8 (55.3; 58.4)
Females	52.7 (50.5; 55.0)	52.1 (50.0; 54.4)	53.6 (51.5; 55.8)	50.8 (48.8; 52.8)	49.5 (47.6; 51.6)
Males	62.0 (59.3; 64.7)	63.0 (60.3; 65.7)	65.7 (63.2; 68.3)	62.9 (60.5; 65.4)	63.9 (61.5; 66.4)
					(1 of 2)

Estimates on incidence proportion of schizophrenia are age- and sex-standardized to the population of Germany on December 31, 2021.

Table S2 (continued): Standardized incidence proportion (with 95% CIs) of schizophrenia by demographic characteristics and calendar year (per 100,000 persons)

	**2017**	**2018**	**2019**	**2020**	**2021**
**Total number of (database) population, 0–64 years, n**	11,615,083	11,846,787	12,155,616	12,349,910	12,450,531
**Overall**					
Both sexes	46.5 (45.2; 47.7)	43.5 (42.3; 44.7)	44.4 (43.2; 45.6)	42.4 (41.3; 43.6)	41.3 (40.1; 42.4)
Females	41.8 (40.2; 43.5)	38.2 (36.7; 39.7)	38.3 (36.8; 39.8)	37.7 (36.3; 39.2)	37.1 (35.6; 38.6)
Males	51.0 (49.1; 52.9)	48.6 (46.8; 50.5)	50.4 (48.6; 52.2)	46.9 (45.2; 48.7)	45.3 (43.6; 47.0)
**Age group 0–17 years**					
Both sexes	3.9 (3.2; 4.7)	4.1 (3.4; 5.0)	4.1 (3.4; 5.0)	4.0 (3.3; 4.9)	3.8 (3.1; 4.7)
Females	3.9 (3.0; 5.2)	3.3 (2.4; 4.5)	3.9 (3.0; 5.2)	4.2 (3.2; 5.6)	4.4 (3.3; 5.7)
Males	3.8 (2.9; 5.0)	4.9 (3.8; 6.3)	4.2 (3.2; 5.5)	3.8 (2.9; 5.1)	3.3 (2.4; 4.5)
**Age group 18–64 years**					
Both sexes	58.1 (56.5; 59.7)	54.2 (52.7; 55.7)	55.4 (53.9; 56.9)	52.8 (51.4; 54.3)	51.5 (50.0; 52.9)
Females	52.0 (50.0; 54.0)	47.6 (45.7; 49.5)	47.5 (45.6; 49.4)	46.7 (44.9; 48.6)	45.9 (44.0; 47.7)
Males	64.0 (61.6; 66.4)	60.7 (58.4; 63.0)	63.1 (60.8; 65.4)	58.8 (56.6; 61.0)	56.9 (54.8; 59.1)
					(2 of 2)

Estimates on incidence proportion of schizophrenia are age- and sex-standardized to the population of Germany on December 31, 2021.

Table S3: Absolute number of persons with incident schizophrenia (“cases”) by sex in 2021

	**Female**	**Male**
**Age group (years)**		
0–12	5	3
13–17	47	38
18–19	59	91
20–21	52	118
22–23	51	129
24–25	65	144
26–29	144	239
30–34	239	357
35–39	248	364
40–44	249	328
45–49	271	199
50–54	308	240
55–59	379	250
60–64	325	206

Table S4: Absolute number persons with schizophrenia (“cases”) by calendar year

	**2012**	**2013**	**2014**	**2015**	**2016**
**Incidence, overall**					
Both sexes	4,427	4,512	5,231	5,200	5,223
Females	2,260	2,262	2,598	2,567	2,497
Males	2,167	2,250	2,633	2,633	2,726
**Incidence, age group 13–17 years**					
Both sexes	85	81	86	91	100
Females	38	41	45	49	47
Males	47	40	41	42	53
**Incidence by urbanicity**[a]					
Large urban city	1,812	1,767	2,100	2,123	2,118
Urban district	1,578	1,673	1,903	1,938	1,874
Rural district (with densification tendencies)	549	588	675	622	665
Sparsely populated rural district	488	483	553	515	566
**Prevalence, overall**					
Females	21,899	22,141	22,091	22,169	21,742
Males	21,582	22,237	22,763	23,128	23,358
					(1 of 2)

[a] Persons with invalid region of residence were excluded.

Table S4 (continued): Absolute number of persons with schizophrenia ("cases") by calendar year

	2017	2018	2019	2020	2021
**Incidence, overall**					
Both sexes	5,436	5,178	5,415	5,253	5,148
Females	2,654	2,452	2,495	2,482	2,442
Males	2,782	2,726	2,920	2,771	2,706
**Incidence, age group 13–17 years**					
Both sexes	92	93	97	95	85
Females	46	36	47	49	47
Males	46	57	50	46	38
**Incidence by urbanicity[a]**					
Large urban city	2,247	2,109	2,151	2,137	2,150
Urban district	1,939	1,920	1,992	1,948	1,828
Rural district (with densification tendencies)	707	631	699	629	641
Sparsely populated rural district	542	518	572	539	529
**Prevalence, overall**					
Females	21,864	21,579	21,405	21,087	20,920
Males	23,690	23,948	24,211	24,488	24,463
					(2 of 2)

[a] Persons with invalid region of residence were excluded.

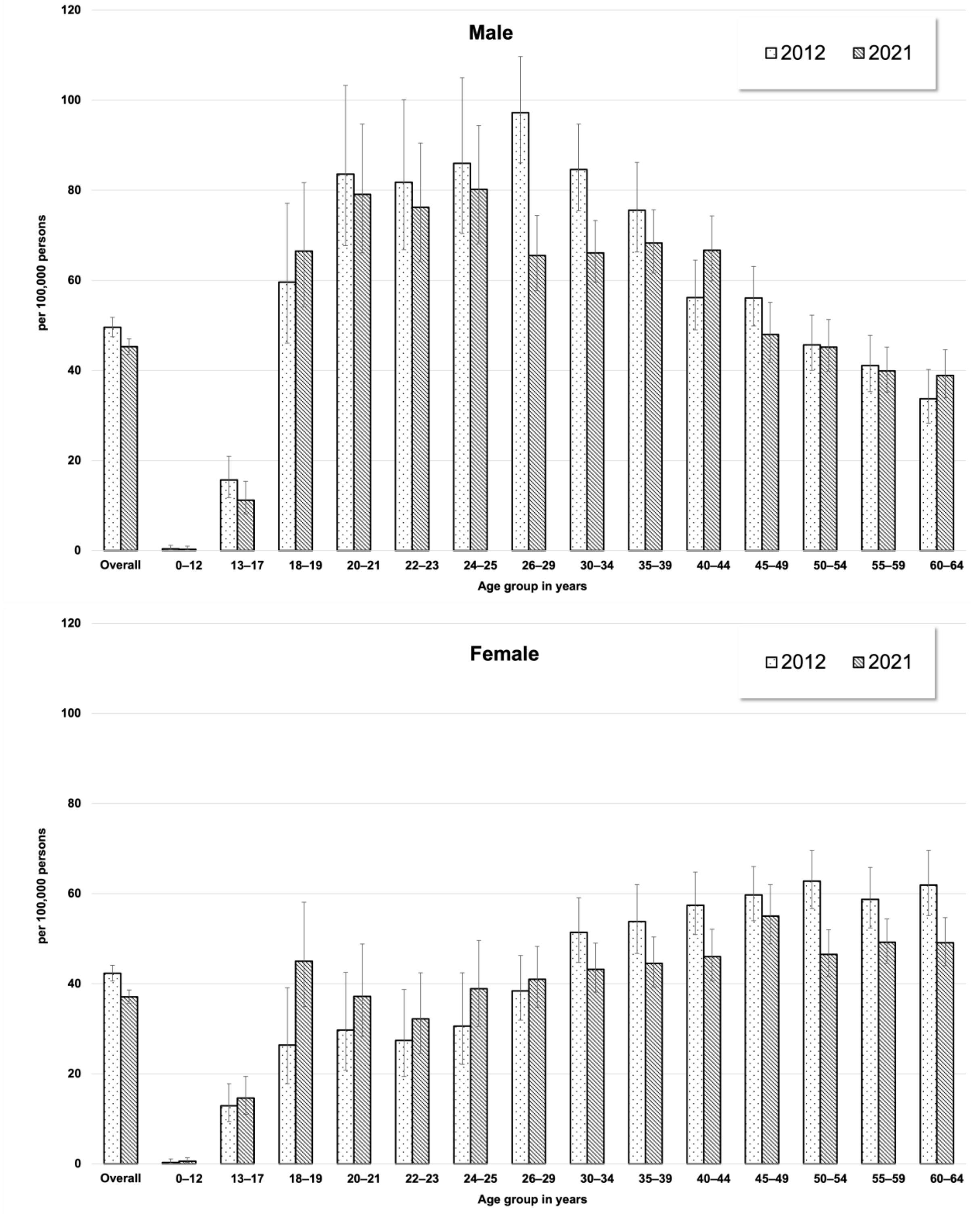

Figure S1: Standardized incidence proportions (with 95% CIs) of schizophrenia in 2012 and 2021 by age among males (upper figure) and females (lower figure) (main analysis = in-/outpatient ICD-10-diagnoses “F20” with antipsychotic treatments)

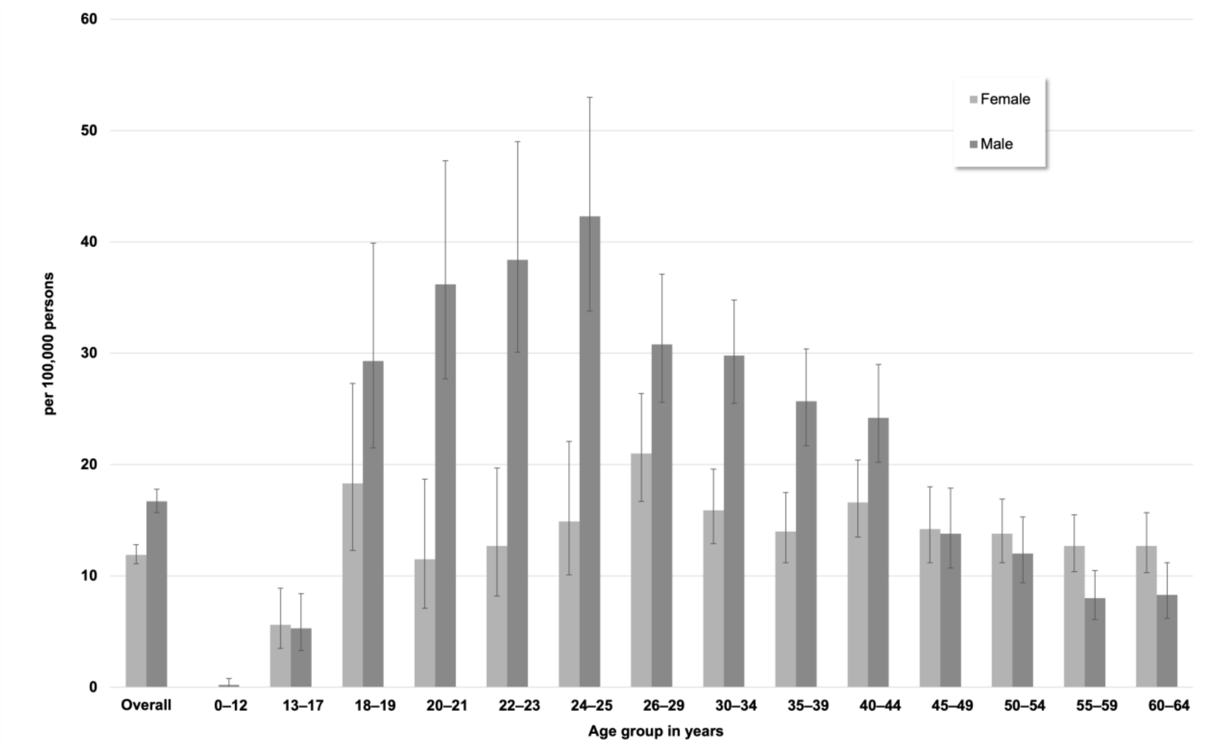

Figure S2: Standardized incidence proportions of schizophrenia (with 95% CIs) by sex and age in 2021 when considering only inpatient ICD-10 “F20” diagnoses with antipsychotic treatments

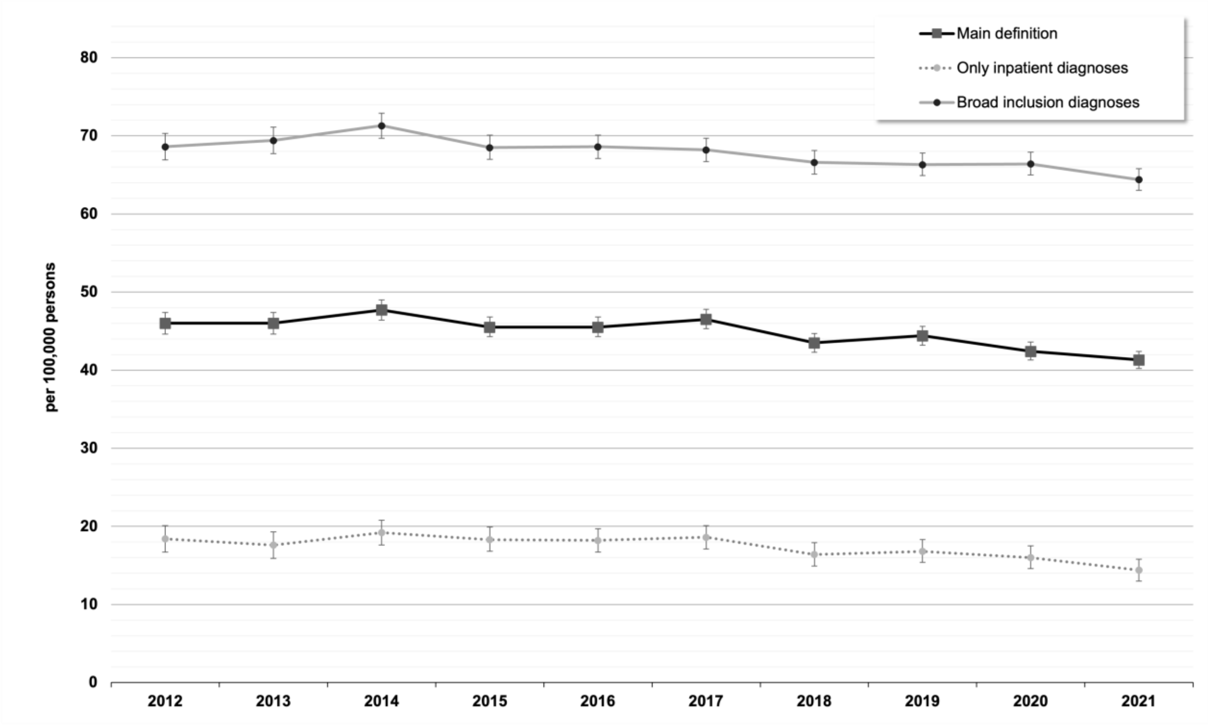

Figure S3: Overall standardized incidence proportion of schizophrenia (with 95% CIs) by calendar year according to (a) the main definition (antipsychotic treatment plus outpatient or inpatient diagnosis F20), (b) when considering only inpatient diagnoses with antipsychotic treatments, or (c) when considering broad inclusion diagnoses (F20, F21, F22, F23, F25, F28, F29) with antipsychotic treatments

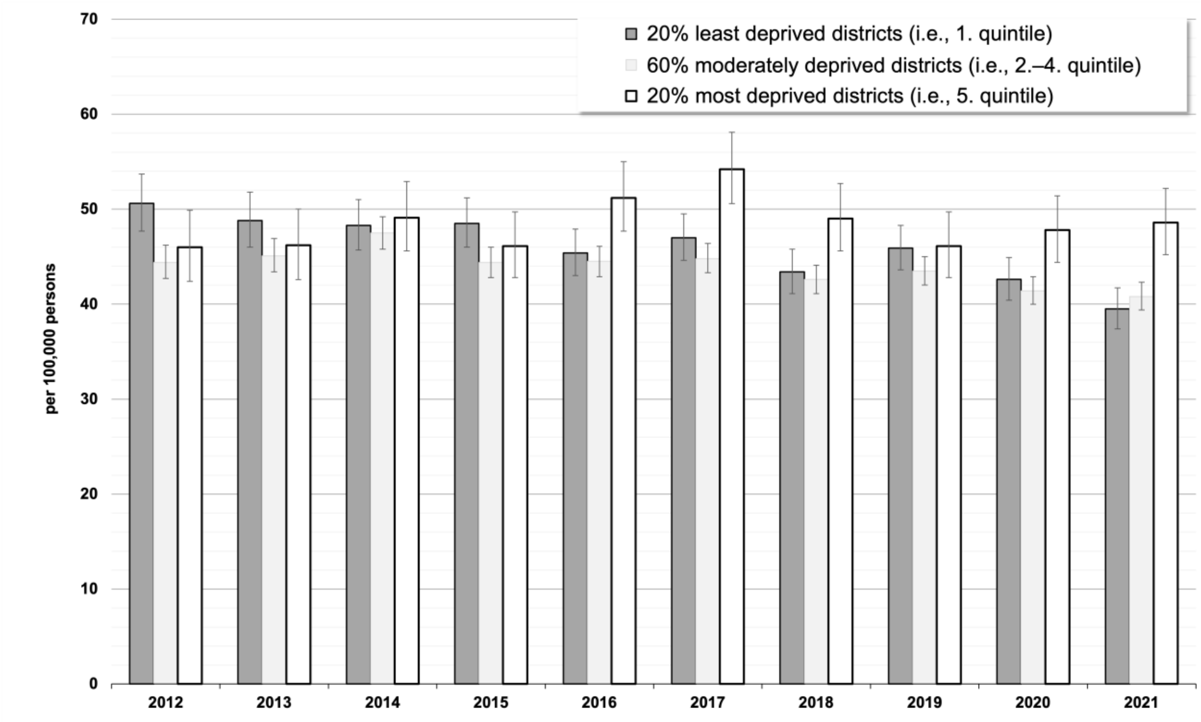

Figure S4: Standardized incidence proportions (with 95% CIs) of schizophrenia by district-level socioeconomic deprivation and calendar year (main analysis = in-/outpatient ICD-10-diagnoses “F20” with antipsychotic treatments)
